# Spectral degree of polarization uniformity for polarization-sensitive OCT

**DOI:** 10.1080/09500340.2014.945501

**Published:** 2014-08-01

**Authors:** Bernhard Baumann, Stefan Zotter, Michael Pircher, Erich Götzinger, Sabine Rauscher, Martin Glösmann, Jan Lammer, Ursula Schmidt-Erfurth, Marion Gröger, Christoph K. Hitzenberger

**Affiliations:** ^a^ Center for Medical Physics and Biomedical Engineering, Medical University of Vienna, Vienna, Austria; ^b^ Medical Imaging Cluster, Medical University of Vienna, Vienna, Austria; ^c^ Core Facility Imaging, Medical University of Vienna, Vienna, Austria; ^d^ Core Facility for Research and Technology, University of Veterinary Medicine Vienna, Vienna, Austria; ^e^ Department of Ophthalmology and Optometry, General Hospital and Medical University Vienna, Vienna, Austria

**Keywords:** optical coherence tomography, polarization-selective devices, multiple scattering, medical and biological imaging

## Abstract

Depolarization of light can be measured by polarization-sensitive optical coherence tomography (PS-OCT) and has been used to improve tissue discrimination as well as segmentation of pigmented structures. Most approaches to depolarization assessment for PS-OCT – such as the degree of polarization uniformity (DOPU) – rely on measuring the uniformity of polarization states using spatial evaluation kernels. In this article, we present a different approach which exploits the spectral dimension. We introduce the spectral DOPU for the pixelwise analysis of polarization state variations between sub-bands of the broadband light source spectrum. Alongside a comparison with conventional spatial and temporal DOPU algorithms, we demonstrate imaging in the healthy human retina, and apply the technique for contrasting hard exudates in diabetic retinopathy and investigating the pigment epithelium of the rat iris.

## 1. Introduction

Polarization-sensitive (PS) optical coherence tomography (OCT) is one of the most promising approaches for extending image contrast in OCT. In addition to imaging based on the intensity of backscattered light, PS-OCT provides access to polarization properties of tissues, thus alleviating discrimination and segmentation of structures. PS-OCT also enables quantitative measurements of quantities such as birefringence, diattenuation, and depolarization [1–3]. While birefringent properties have been successfully assessed in a growing variety of tissues (retinal nerve fiber layer, muscles and tendons, and aortic plaques) [4–6], depolarization has only recently been started to be exploited with PS-OCT [7,8].

Depolarization of signals in PS-OCT refers to the randomization of the incident light’s polarization state by processes such as scattering by randomly oriented ellipsoid particles with sizes comparable to the wavelength of light [9], or multiple scattering [8,10]. In general, in order to assess depolarization in PS-OCT images, the uniformity of polarization states has been analyzed in areas or volumes covering several image pixels. Depolarization is characterized by a decorrelation of polarization states within small scan regions and hence by a strong variation of polarization states within the respective evaluation kernel. Different metrics have been developed to cast the uniformity of Stokes vectors in Poincaré space to a clear and comprehensible quantity [7,8,11,12]. Of those, the degree of polarization uniformity (DOPU) will be considered for the present investigation [7]. In a nutshell, DOPU gives the length of the average Stokes vector [*Q*, *U*, *V*]^*T*^ in the evaluation window. The more uniform the orientation of the vectors, the closer DOPU will be to 1. Conversely, in case of depolarization, lower DOPU values will be computed. For the evaluation kernel, two- and three-dimensional windows have been used [7,13], along with the temporal approaches where the Stokes vector elements were averaged among repeatedly acquired images [14].

In this article, we demonstrate an alternative approach to depolarization assessment with PS-OCT. In contrast to previous work, which focused on analyzing the spatial variation of polarization states, we propose to exploit the broad spectral bandwidths inherent to OCT measurements and evaluate depolarization between different spectral components at the same spatial location.

## 2. Spectral DOPU

The measurement of Stokes vectors in PS-OCT is generally based on the acquisition of two orthogonal polarization components *E*_1_ and *E*_2_. From the amplitudes *A*_1,2_ of and the phase difference ΔΦ between *E*_1_ and *E*_2_, the Stokes vector

(1) $${\left[I,Q,U,V\right]}^{T}={\left[A_{1}^{2}+A_{2}^{2},A_{1}^{2}-A_{2}^{2},\;2A_{1}A_{2}\cos\Delta \Phi ,\;2A_{1}A_{2}\sin\Delta \Phi \right]}^{T}$$

can be computed for every image pixel. Conventional (spatial) DOPU is calculated from averaged and normalized Stokes vector elements in a kernel as $\sqrt{{\langle Q\rangle }^{2}+{\langle U\rangle }^{2}+{\langle V\rangle }^{2}},$ where the angle brackets denote spatial average. For a single image pixel, DOPU corresponds to the well-known degree of polarization (DOP) and equals 1. Similarly, DOPU = 1 holds for an ensemble of linearly dependent (i.e. parallel) Stokes vectors [*Q*, *U*, *V*]^*T*^ in polarization preserving tissue, and DOPU is significantly lower in case of depolarization.

The polarization state of light after scattering processes depends not only on the optical parameters of the sample but also on the wavelength of light [15]. Therefore, it makes sense to investigate polarization effects for different spectral regions of backscattered light as pointed out by de Boer and Milner [2]. In Fourier-domain PS-OCT, the spectral interferogram *s*(*k*,*x*) is readily available which makes it possible to access the polarization states for sub-bands of the light source spectrum for every image pixel.

The orthogonal polarization components of the spectral interferogram, *s*_1,2_(*k*) are functions of sample and reference arm reflectivity *R*_*S*,*R*_(*k*), the source spectrum *S*(*k*), the detection efficiency *η*(*k*), and the polarization-dependent weight *e*_1,2_(*k*): $[s_{1,2}\left(k\right)=\sqrt{R_{S}\left(k\right)R_{R}\left(k\right)}S\left(k\right)\;\eta \left(k\right)\;e_{1,2}\left(k\right)=\tilde{S}\left(k\right)\;e_{1,2}\left(k\right)$. The Jones vector components *E*_1,2_ are computed by inverse Fourier transform of *s*_1,2_, $E_{1,2}\left(z\right)=FT^{-1}\left\{s_{1,2}\left(k\right)\right\}$. Therefore, *E*_1,2_ and thus [*Q*,*U*,*V*]^*T*^ represent the average polarization state in the position ***r*** = [*x*, *y*, *z*], spectrally weighted by *e*_1,2_. However, the polarization states of sub-bands of *S*(*k*) can be found by applying a window *G*(*k*_*β*_, Δ*k*) prior to the Fourier transform, $E_{1,2}^{\beta }\left(z\right)=FT^{-1}\left\{G\left(k_{\beta },\Delta k\right)\cdot s_{1,2}\left(k\right)\right\}$, where *k*_*β*_ and Δ*k* are the central wavenumber and the bandwidth of spectral band *β*. In order to measure the uniformity of polarization states across different spectral components *β* in a single image pixel, we now define the spectral DOPU

(2) $$sDOPU=\frac{\sqrt{{{\langle Q\rangle }_{\beta }}^{2}+{{\langle U\rangle }_{\beta }}^{2}+{{\langle V\rangle }_{\beta }}^{2}}}{{\langle I\rangle }_{\beta }}$$

where ${\langle \;\cdot \;\rangle }_{\beta }$ denotes the average of the Stokes parameters across all spectral bands. Analogous to the conventional (spatial) DOPU, sDOPU values will equal unity only when the averaged Stokes vectors are collinear. If polarization states differ between the spectral sub-bands, sDOPU will drop accordingly.

## 3. Experimental setups

Spectral-domain PS-OCT systems based on bulk- [16] and fiber-optic [14] interferometers, respectively, were used to demonstrate the concept of spectral DOPU imaging. Both instruments employed superluminescent diodes (Superlum Diodes, Inc.) at ~840 nm wavelength with spectral widths of ~60 and ~50 nm (FWHM), respectively. Circularly polarized light was used for sample illumination. At the interferometer exit, the OCT signal was split into its S and P polarized components, which were detected by two identical spectrometer units. The spectrometer line scan cameras were operated at 20 and 70 kHz line rates, respectively.

## 4. Results

**Figure 1.**
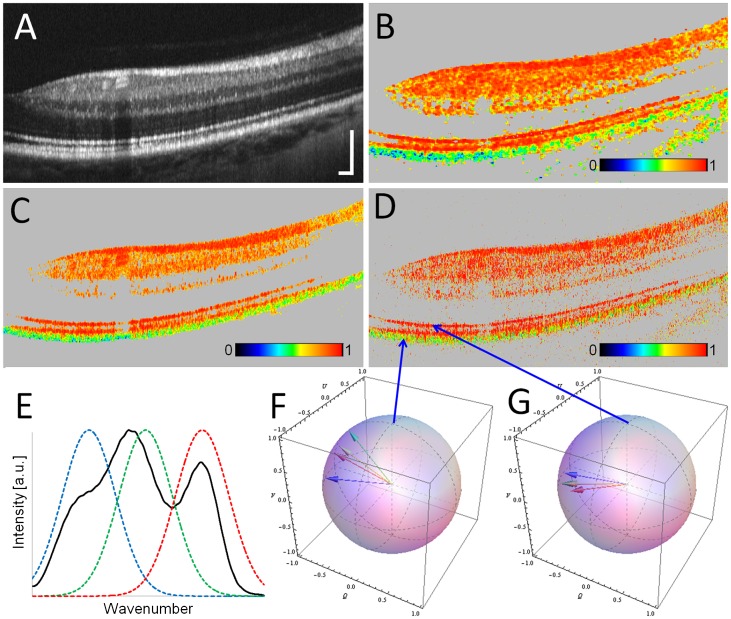
Depolarization imaging in healthy human retina. (*A*) Reflectivity B-scan image (average of 30 frames). Scale bars: 250 μm. (*B*) Spatial DOPU image. (*C*) Temporal DOPU image. (*D*) sDOPU image. (*E*) Spectrum (black) and Gaussian windows (blue, green, and red). (*F*)–(G) Poincaré sphere representation of Stokes vectors in depolarizing RPE (*F*) and polarization preserving photoreceptors (*G*). (The colour version of this figure is included in the online version of the journal.)

In order to compare the different methods for DOPU assessment, a healthy subject’s maculopapillary retina was scanned with the fiber-based PS-OCT system. An average reflectivity image of a sequence of 30 B-scans recorded in the same position over 0.43 seconds is shown in Figure 1(*A*). Axial (*z*) and transverse (*x*) eye motion amounted to 15 and 14 μm (standard deviation), respectively, and were compensated prior to averaging by cross-correlating and aligning successive frames. Figure 1(*B*) shows a standard (spatial) DOPU image of a single frame with an evaluation window of 8 (*x*) × 5 (*z*) pixels (~47 μm × 9 μm). Depolarization can be observed in the retinal pigment epithelium (RPE) as low DOPU values, while the reddish color of the neural retina indicates rather uniform polarization states, i.e. DOPU values close to 1. Pixels with low reflectivity values are displayed in gray. A temporal DOPU image was computed by averaging the Stokes parameters for each image pixel over the image sequence (Figure 1(*C*)). In Figure 1(*D*), a spectral DOPU image computed from the frame used also in 1(*B*) is shown. The detected spectrum was decomposed into three sub-bands using Gaussian envelopes centered at ~820, 840, and 860 nm with spectral FWHM widths of ~20 nm (Figure 1(*E*)). Stokes vectors were computed for each sub-band and averaged to compute sDOPU according to Eq. (2) for each pixel. Stokes vectors for an image pixel in the depolarizing RPE and in the polarization preserving photoreceptor layer are represented in Poincaré spheres in Figure 1(*F*) and (*G*), respectively. Clearly, the Stokes vectors of the three sub-bands plotted in blue, green, and red align quite well in the photoreceptor layer whereas they branch out in the RPE.

**Figure 2.**
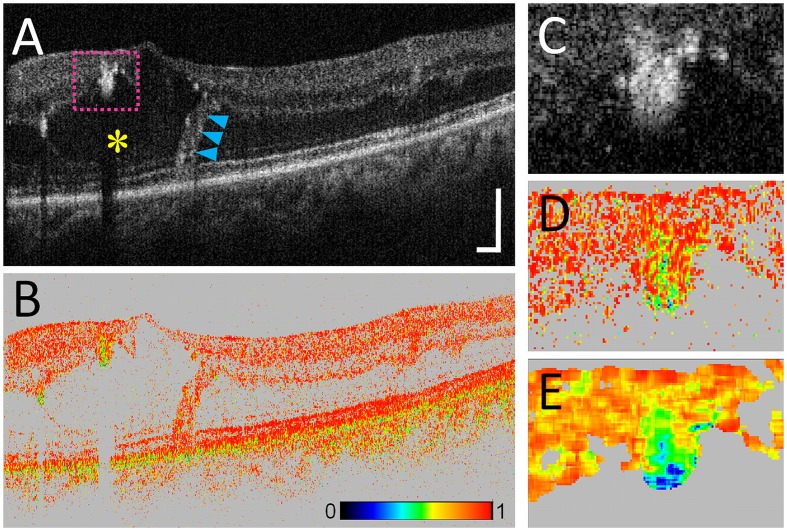
Imaging depolarizing pathological lesions in diabetic retinopathy. (*A*) Reflectivity B-scan image revealing edema (asterisk), structure caused by laser therapy (arrow heads), and hard exudate (box). (*B*) sDOPU image indicating depolarization in RPE and hard exudates. Right column shows magnified views of the box in (*A*): (*C*) Reflectivity. (*D*) sDOPU. (*E*) Spatial DOPU (window: 8 (*x*) × 5 (*z*) pixels). Scale bars: 250 μm. (The colour version of this figure is included in the online version of the journal.)

Spectral DOPU imaging was also applied for investigating pathological features in the retina of a patient with diabetic retinopathy, who was imaged with the bulk optics-based PS-OCT system (Figure 2). In the reflectivity image (Figure 2(*A*)), disruptions of the regular retinal structure by an edema and laser scars are visible. Hyperscattering hard exudates – deposits of lipoproteins framing the edema lesion – can be observed (Figure 2(*C*)). In the spectral DOPU image (Figure 2(*B*)), these exudates appear depolarizing – similar to the RPE. However, while in the RPE polarization scrambling is accounted to the melanin granules [17], the cause of depolarization in hard exudates still requires more investigations. A comparison between spectral and spatial DOPU images of the prominent exudates reveals generally lower DOPU values and – due to the 8 × 5 pixel kernel – a more smeared out appearance for the spatial evaluation (Figure 2(*E*)). Conversely, the DOPU image appears more speckled for the spectral evaluation (Figure 2(*D*)).

**Figure 3.**
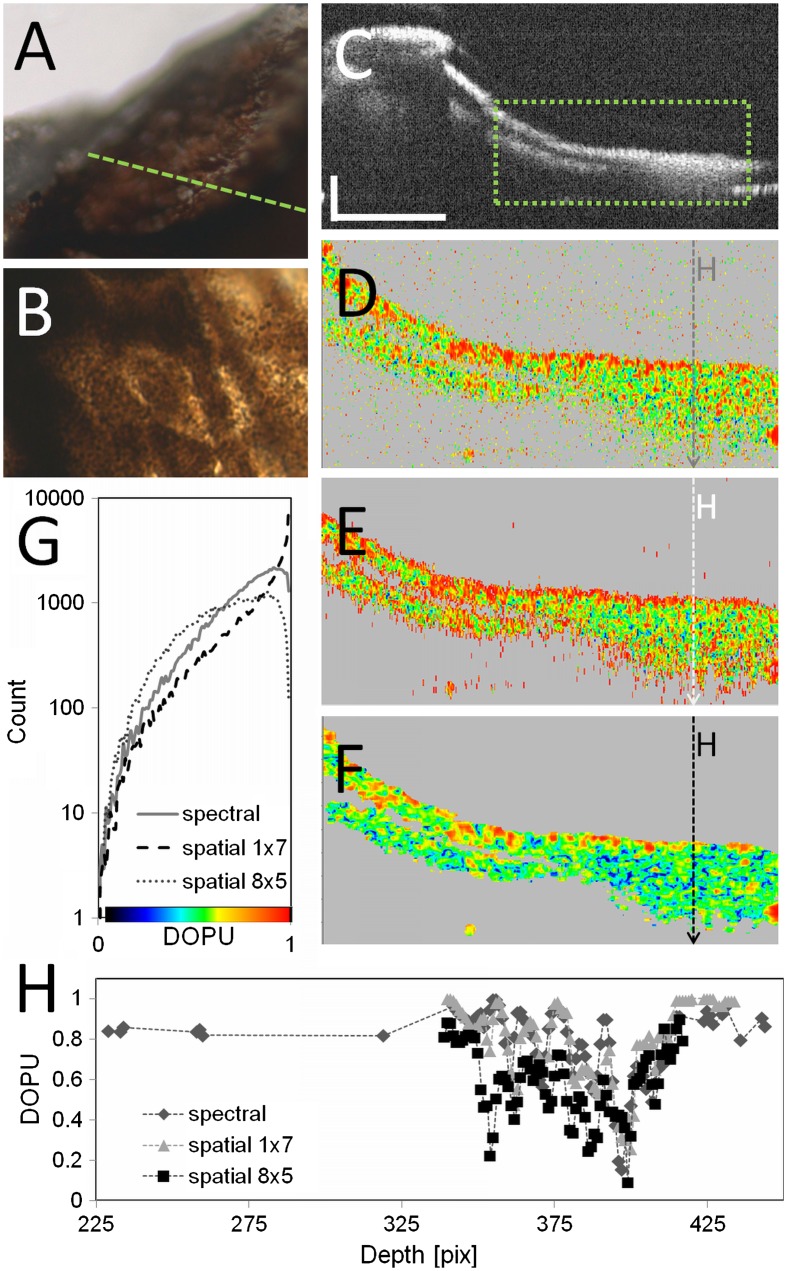
Rat iris imaged at the site of the pigment epithelium. (*A*) Photomicrograph. (*B*) Confocal microscopy image revealing individual melanin granules. (*C*) Reflectivity B-scan image. Green rectangle indicates the position of the DOPU images. Scale bars: 250 μm. (*D*) sDOPU image. (*E*) Spatial DOPU image (kernel: 1 × 7 pixels). (*F*) Spatial DOPU image (kernel: 5 × 8 pixels). (*G*) Histogram of DOPU values in (*D*)–(*F*). (H) Depth profiles at locations indicated by the arrow in the DOPU images (*D*)–(*F*). (The colour version of this figure is included in the online version of the journal.)

Imaging of a sample with strong scattering and depolarization was performed in the puckered iris of an adult pigmented rat (Long Evans, ex vivo). Figure 3(*A*) shows a photomicrograph of the imaged portion of the iris. The dark brown appearance is due to the melanin pigmentation of the posterior iris epithelium. Single melanin granules can be observed in a confocal microscopy image (Figure 3(*B*)). PS-OCT images acquired with the fiber-based system show high absorption and strong scatter alongside a fuzzy look in the reflectivity image (Figure 3(*C*)). DOPU images were calculated using Eq. (2) (Figure 3(*D*)), and using a spatial kernel of 1 × 7 (~1 μm × 11 μm, Figure 3(*E*)) and 8 × 5 pixels (~8 μm × 8 μm, Figure 3(*F*)), respectively. All images exhibit polarization preserving characteristics at the surface and strong depolarization within the sample. Histograms of the DOPU values in Figure 3(*D*)–(*F*) were computed in the dashed box indicated in Figure 3(*C*) and are shown in Figure 3(*G*). Further, depth profiles are shown in Figure 3(*H*) for easy comparison. Similar to the imagery in Figures 1 and 2, a shift towards lower DOPU values can be observed for the larger spatial window. This result is expected since a larger number of speckles – and hence a larger number of different polarization states – are covered by the larger window.

## 5. Discussion and conclusion

An alternative approach for depolarization assessment in PS-OCT was developed. In contrast to previous DOPU methods using spatial or temporal averaging of Stokes vector elements, we chose a spectral-domain approach. In order to compare the results from the different approaches, we analyzed the same PS-OCT raw data using spatial, temporal, and spectral DOPU. As shown in Figure 1, the appearance of the retinal structures is similar but not identical for the different methods. The greatest reduction of spatial resolution can be observed in Figure 1(*B*), where a spatial DOPU kernel of 8 (*x*) × 5 (*z*) pixels acts as a rectangular smoothing filter. Less-pronounced depolarization, i.e. in general higher DOPU values, is visible in Figure 1(*C*), where a temporal DOPU approach was employed. Temporal averaging relies on small movements of the sample (or patient) between successive frames. For the 30 frames used for temporal DOPU calculation in Figure 1(*C*), in-plane eye motion of ~15 μm was observed in both *x* and *z* direction. While in-plane eye motion was compensated prior to averaging respective Stokes vector elements, the (unknown) motion orthogonal to the frame essentially leads to averaging and DOPU computation in *y*-direction. Assuming *y*-motion of ~15 μm analogous to in-plane motion, the number of speckles contributing to each DOPU pixel in Figure 1(*B*) is lower than for the spatial approach (Figure 1(*B*)). Therefore, it is not surprising that DOPU values are in general higher in Figure 1(*C*), while the speckle size appears smaller. The spectral DOPU approach (Figure 1(*D*)) leads to an even more red-shifted appearance with even higher DOPU values and even less speckle blur. For sDOPU, only three Stokes vector elements – one for each spectral band – were averaged for each image pixels. The depolarizing RPE is well discernible from the non-depolarizing neural retina.

In addition to the RPE in a healthy subject (Figure 1), we also demonstrated sDOPU imaging in diabetic maculopathy (Figure 2). Hard exudates surrounding edema lesions were observed as depolarizing structures in spatial and spectral DOPU images. Recently, we demonstrated the value of PS-OCT for imaging and segmentation of such exudates in the diabetic retina [18]. Therefore, the sDOPU approach presented here might be interesting for the future development of automatic segmentation algorithms for the detection of hard exudates as well as their precursors, i.e. small exudative microfoci [19].

In this article, we used three spectral bands (*N*_*β*_ **= 3). In principle, the number of spectral bands could be chosen anywhere in [1, *N*], *N* being the number of spectral sampling points. The limit *N*_*β*_ **= 1 corresponds to the classical calculation of Stokes parameters for polychromatic light [20], which in case of PS-OCT would always be unity. For large *N*_*β*_ and using a narrow spectral window, the Stokes parameters *Q*_*i*_ etc. for each window *i* can be linked to their Stokes spectral functions, $Q_{i}^{\prime }=Q_{i}/\Delta k$ etc. Then, for sufficiently large *N*_*β*_, the averaged Stokes parameters approach the integrated spectral Stokes parameters ($\overset{¯}{Q}=\int_{0}^{\infty }Q^{\prime }\left(k\right)dk$ etc.) [21]. As does the number of spectral windows *N*_*β*_, also the shape *G*(*k*_*β*_, Δ*k*) impacts the outcome of sDOPU computation. When $G\overset{FT}{⟷}g$, the spectral average of *Q* (and likewise *U* and *V*) corresponds to a spatial average in a *g*-shaped evaluation kernel. Notably, in this particular case, sDOPU is equivalent to DOPU computed for a kernel of 1 pixel (*x*) × *g* (*z*). The choice of dedicated windows in the spectral-domain may alleviate the implementation of tailored evaluation kernels which could otherwise be challenging using a conventional spatial DOPU approach.

The size of the evaluation window influences the number of speckles investigated for DOPU calculation. In this paper, linear windows were compared for spectral and spatial DOPU computation, for instance, in Figure 3(*D*) and (*E*). Such windows preserve rather high lateral image resolution. When a 2D or 3D DOPU computation kernel is used, a larger number of speckles can be evaluated – however, at the expense of image resolution. Depending on the application, sDOPU computation could also be combined with spatial 2D or 3D evaluation kernels or with temporal averaging. sDOPU may be useful for multispectral OCT approaches using several light sources, for spectroscopic tissue imaging, and scattering analysis.

In conclusion, the results shown in this article demonstrate a DOPU method based on analyzing the uniformity of polarization states among spectral sub-bands. The method was applied to imaging depolarizing tissues in healthy and diseased human retinas as well as in ex vivo rat iris. Analogous to conventional DOPU methods, the approach has the potential to strengthen and improve the segmentation of biological tissues and pathologic lesions. Moreover, knowledge of spectral polarization characteristics may be of particular interest for the analysis of processes such as multiple scattering.
